# Bromination Pattern of Hydroxylated Metabolites of BDE-47 Affects Their Potency to Release Calcium from Intracellular Stores in PC12 Cells

**DOI:** 10.1289/ehp.0901339

**Published:** 2009-11-19

**Authors:** Milou M.L. Dingemans, Harm J. Heusinkveld, Åke Bergman, Martin van den Berg, Remco H.S. Westerink

**Affiliations:** 1 Neurotoxicology Research Group, Toxicology Division, Institute for Risk Assessment Sciences, Utrecht University, Utrecht, the Netherlands; 2 Department of Environmental Chemistry, Stockholm University, Stockholm, Sweden

**Keywords:** brominated flame retardant, calcium, calcium fluctuations, Fura-2, intracellular calcium stores, neurotoxicity, PC12, persistent organic pollutant, polybrominated diphenyl ether, structure–activity relationship

## Abstract

**Background:**

Brominated flame retardants, including the widely used polybrominated diphenyl ethers (PBDEs), have been detected in humans, raising concern about possible neurotoxicity. Recent research demonstrated that the hydroxylated metabolite 6-OH-BDE-47 increases neurotransmitter release by releasing calcium ions (Ca^2+^) from intracellular stores at much lower concentrations than its environmentally relevant parent congener BDE-47. Recently, several other hydroxylated BDE-47 metabolites, besides 6-OH-BDE-47, have been detected in human serum and cord blood.

**Objective and Methods:**

To investigate the neurotoxic potential of other environmentally relevant PBDEs and their metabolites, we examined and compared the acute effects of BDE-47, BDE-49, BDE-99, BDE-100, BDE-153, and several metabolites of BDE-47—6-OH-BDE-47 (and its methoxylated analog 6-MeO-BDE-47), 6′-OH-BDE-49, 5-OH-BDE-47, 3-OH-BDE-47, and 4′-OH-BDE-49—on intracellular Ca^2+^ concentration ([Ca^2+^]*_i_*), measured using the Ca^2+^-responsive dye Fura-2 in neuroendocrine pheochromocytoma (PC12) cells.

**Results:**

In contrast to the parent PBDEs and 6-MeO-BDE-47, all hydroxylated metabolites induced Ca^2+^ release from intracellular stores, although with different lowest observed effect concentrations (LOECs). The major intracellular Ca^2+^ sources were either endoplasmic reticulum (ER; 5-OH-BDE-47 and 6′-OH-BDE-49) or both ER and mitochondria (6-OH-BDE-47, 3-OH-BDE-47, and 4′-OH-BDE-49). When investigating fluctuations in [Ca^2+^]*_i_*, which is a more subtle end point, we observed lower LOECs for 6-OH-BDE-47 and 4′-OH-BDE-49, as well as for BDE-47.

**Conclusions:**

The present findings demonstrate that hydroxylated metabolites of BDE-47 cause disturbance of the [Ca^2+^]*_i_*. Importantly, shielding of the OH group on both sides with bromine atoms and/or the ether bond to the other phenyl ring lowers the potency of hydroxylated PBDE metabolites.

Brominated flame retardants are added to a wide array of consumer products. Adverse effects of these compounds on the developing nervous system give cause for concern (for review, see Costa and Giordano 2007). Long-lasting neurobehavioral changes after neonatal exposure have been detected in rodents after exposure to polybrominated diphenyl ethers (PBDEs) (Branchi et al. 2003; Eriksson et al. 2001; Lilienthal et al. 2006; Rice et al. 2007; Viberg et al. 2003, 2006), as well as various functional and structural alterations in the brain (Dingemans et al. 2007; Viberg 2009; Xing et al. 2009).

During development, acute effects on neuronal activity may result in long-lasting changes in proteins, brain function, and behavior. A key regulator for neuronal function is intracellular concentration of calcium ions, [Ca^2+^]*_i_*, which regulates many cellular processes, including the release of neurotransmitters at the presynaptic terminal (for reviews, see Clapham 2007; Laude and Simpson 2009).

PBDEs have been shown to affect Ca^2+^ homeostasis in microsomes (Kodavanti and Ward 2005) and pheochromocytoma (PC12) cells (Dingemans et al. 2007) and to reduce the Ca^2+^ uptake by brain microsomes and mitochondria (Coburn et al. 2008) at relatively high concentrations. PBDEs have also been shown to induce oxidative stress in neuronal cells (He et al. 2008; Kodavanti and Ward 2005). Recently, 6-OH-BDE-47 (6-hydroxy-2,2′,4,4′-tetrabromodiphenyl ether), a hydroxylated metabolite of BDE-47 (2,2′,4,4′-tetrabromodiphenyl ether), was demonstrated to increase vesicular neurotransmitter release and [Ca^2+^]*_i_* by releasing Ca^2+^ from intracellular stores in PC12 cells. The increase in vesicular neurotransmitter release was induced by 6-OH-BDE-47 at much lower concentrations than by the parent PBDE (Dingemans et al. 2008). Therefore, hydroxylated PBDEs (OH-PBDEs) may be more important than the parent compounds for human risk assessment for neurotoxicity.

Recently, not only PBDEs but also various hydroxylated metabolites of PBDEs have been found to bioaccumulate in humans (Athanasiadou et al. 2008; Qiu et al. 2009). Therefore, the aim of the present study was to determine whether monohydroxylated metabolites of the abundant BDE-47 affect [Ca^2+^]*_i_* and to compare this with the effects of a methoxylated analog and several other environmentally relevant PBDE congeners.

## Materials and Methods

### Chemicals

In the present study, we investigated the effects of BDE-47, BDE-99 (2,2′,4,4′,5-pentabromodiphenyl ether), BDE-100 (2,2′,4,4′,6-pentabromodiphenyl ether), and BDE-153 (2,2′,4,4′,5,5′-hexabromodiphenyl ether), the main constituents of the commercial DE-71 pentaBDE mixture (La Guardia et al. 2006), as well as several metabolites of BDE-47 [6-OH-BDE-47, 6′-OH-BDE-49 (6′-hydroxy-2,2′,4,5′-tetrabromodiphenyl ether), 5-OH-BDE-47 (5-hydroxy-2,2′,4,4′-tetrabromodiphenyl ether), 3-OH-BDE-47 (3-hydroxy-2,2′,4,4′-tetrabromodiphenyl ether), 4′-OH-BDE-49 (4′-hydroxy-2,2′,4,5′-tetrabromodiphenyl ether), and the methoxylated analog 6-MeO-BDE-47 (6-methoxy-2,2′,4,4′-tetrabromodiphenyl ether)]. During formation of 6′-OH-BDE-49 and 4′-OH-BDE-49, a bromine (Br)-shift takes place. Consequently, we included BDE-49 (2,2′,4,5′-tetrabromodiphenyl ether) as a control for possible influences of this change in bromination pattern.

**Figure 1:**
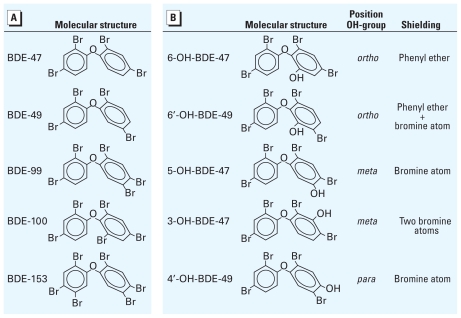
Molecular structures of (*A*) the PBDEs (BDE-47, BDE-49, BDE-99, BDE-100, and BDE-153) and (*B*) the metabolites of BDE-47 (6-OH-BDE-47, 6′-OH-BDE-49, 5-OH-BDE-47, 3-OH-BDE-47, and 4′-OH-BDE-49) investigated in this study. For the metabolites, the position of the OH group is indicated, as well as the shielding of this group by occupancy of the adjacent carbon atoms by either phenyl ether and/or Br atoms.

PBDEs (Figure 1) [see also Supplemental Material, Table 1 (doi:10.1289/ehp.0901339)] were synthesized and purified (~ 99% purity) at the Department of Environmental Chemistry, Stockholm University (Marsh et al. 1999). Dibenzo-*p*-dioxins and dibenzofurans were removed from the PBDEs with a charcoal column as described by Örn et al. (1996). All other chemicals, unless otherwise stated, were obtained from Sigma-Aldrich (Zwijndrecht, the Netherlands).

### Cell culture

Rat pheochromocytoma (PC12) cells (Greene and Tischler 1976), obtained from ATCC (American Type Culture Collection, Manassas, VA, USA), were cultured as described previously [Dingemans et al. 2008; see also Supplemental Material (doi:10.1289/ehp.0901339)].

### Cell viability assay

To investigate possible acute effects of the PBDEs on cell viability, we used the alamarBlue (AB) and neutral red (NR) uptake assays with minor modifications [Magnani and Bettini 2000; Repetto et al. 2008; see also Supplemental Material (doi:10.1289/ehp.0901339)].

### Intracellular Ca^2+^ imaging

Changes in [Ca^2+^]*_i_* were measured using the Ca^2+^-sensitive fluorescence ratio dye Fura-2 as described previously (Dingemans et al. 2007, 2008). For detailed information on experimental conditions and calculation of [Ca^2+^]*_i_*, see Supplemental Material (doi:10.1289/ehp.0901339).

The average and amplitude of [Ca^2+^]*_i_* during exposure were determined per cell to investigate effects of PBDEs on Ca^2+^ homeostasis. The standard deviation during baseline [Ca^2+^]*_i_* recording ranged from 2% to 74% of average [Ca^2+^]*_i_* [*n* = 1,538; see Supplemental Material, Figure 1 (doi:10.1289/ehp.0901339)]. To prevent registration of false-positive effects, an increase in [Ca^2+^]*_i_* to > 175% of baseline was used to determine no observed effect concentrations (NOECs) and lowest observed effect concentrations (LOECs). A transient increase within 0–10 min from the start of exposure is referred to as an “initial increase,” whereas additional increases are referred to as “late increases” (Dingemans et al. 2008). At NOEC levels, based on average and amplitude of [Ca^2+^]*_i_* levels, increases in [Ca^2+^]*_i_* levels to > 175% of baseline during exposure were scored as fluctuations to investigate more subtle effects on Ca^2+^ homeostasis. We determined the number of cells showing fluctuations in [Ca^2+^]*_i_*, as well as frequency, amplitude, and duration of these fluctuations.

### Data analysis and statistics

All data are presented as mean ± SE from the number of cells or fluctuations in [Ca^2+^]*_i_*. We performed statistical analyses using SPSS 16 (SPSS, Chicago, IL, USA). Categorical and continuous data were compared using Fisher’s exact test and Student’s *t*-test, respectively, paired or unpaired where applicable. Analysis of variance (ANOVA) and post hoc *t*-tests were performed to investigate possible dose–response relationships. To investigate structure–activity relationships, we investigated the possible influence of hydroxylation position and/or shielding of the OH group by adjacent atomic groups on the efficacy to increase [Ca^2+^]*_i_*. To this aim, we performed a multifactorial ANOVA using the mean increase in basal [Ca^2+^]*_i_* induced by 20 μM of the different OH-PBDEs as the dependent variable. Hydroxylation position (*ortho*, *meta*, or *para*) and the presence of either one or two shielding atomic groups (phenyl ring and/or Br atom) adjacent to the OH group were used as fixed variables (Figure 1). A *p*-value < 0.05 was considered statistically significant.

## Results

### Effects of parent PBDEs on [Ca^2+^]_i_

Exposure to the solvent control [dimethyl sulfoxide (DMSO)] or to 20 μM of BDE-47, BDE-49, BDE-99, BDE-100, or BDE-153 did not decrease cell viability (data not shown) or increase the average or amplitude of [Ca^2+^]*_i_* or the percentage of cells showing fluctuations in [Ca^2+^]*_i_* [see Supplemental Material, Figure 2 (doi:10.1289/ehp.0901339)]. However, the frequency and duration of fluctuations in [Ca^2+^]*_i_* increased during exposure to 20 μM BDE-47. At 2 μM BDE-47, similar effects were observed (NOEC, 1 μM). No effects could be detected on the frequency, duration, or amplitude of fluctuations during exposure to 20 μM BDE-49, BDE-99, BDE-100, or BDE-153 [see Supplemental Material, Table 3 (doi:10.1289/ehp.0901339)].

### Hydroxylated BDE-47 metabolites increase [Ca^2+^]_i_

After 20 min exposure to 6-OH-BDE-47, 5-OH-BDE-47, or 4′-OH-BDE-49, the NR assay indicated a decrease in cell viability only at the 20-μM dose, with means ± SEs of 86 ± 0.3%, 86 ± 1.1%, and 93 ± 0.8% of control, respectively. We observed no significant decreases in cell viability measured by the NR assay for 6′-OH-BDE-49 or 3-OH-BDE-47. In the AB assay, exposure to 6-OH-BDE-47, 6′-OH-BDE-49, or 3-OH-BDE-47 increased the relative fluorescence intensity dose-dependently above the control level (data not shown).

**Figure 2:**
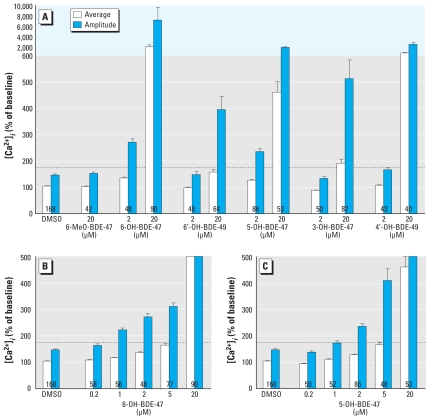
Average and amplitude of [Ca^2+^]*_i_* in PC12 cells exposed to hydroxylated metabolites of BDE-47. (*A*) Cells were exposed to 2 or 20 μM 6-MeO-BDE-47, 6-OH-BDE-47, 6′-OH-BDE-49, 5-OH-BDE-47, 3-OH-BDE-47, or 4′-OH-BDE-49; note the change in scale. We tested additional concentrations of 6-OH-BDE-47 (*B*) and 5-OH-BDE-47 (*C*) because the 2-μM dose of these metabolites increased [Ca^2+^]*_i_* to > 175% of baseline (dashed line). Data are shown from 4–19 experiments per concentration; numbers inside the bars indicate the number of cells used for data analysis.

**Figure 3:**
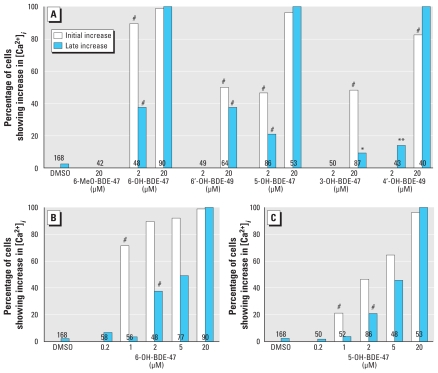
Concentration dependence of the occurrence of different types of [Ca^2+^]*_i_* disturbances in PC12 cells during exposure to 2 or 20 μM of hydroxylated metabolites of BDE-47; values shown are the percentage of cells showing an initial transient increase or a late increase in [Ca^2+^]*_i_*. (*A*) Cells were exposed to 6-MeO-BDE-47, 6-OH-BDE-47, 6′-OH-BDE-49, 5-OH-BDE-47, 3-OH-BDE-47, or 4′-OH-BDE-49. We tested additional concentrations of 6-OH-BDE-47 (*B*) and 5-OH-BDE-47 (*C*) because the 2-μM dose of these metabolites showed initial and late increases of [Ca^2+^]*_i_*. Data are from 4–19 experiments per concentration; numbers inside the bars indicate the number of cells used for data analysis. *p < 0.05, **p < 0.01, and #*p* < 0.001; significant changes in the percentage of cells showing initial or late increases in [Ca^2+^]*_i_* are indicated at the LOEC.

Initial increases in [Ca^2+^]*_i_* were observed during exposure to 6-OH-BDE-47, 6′-OH-BDE-49, 5-OH-BDE-47, 3-OH-BDE-47, or 4′-OH-BDE-49 [see Supplemental Material, Figure 3 and Table 2 (doi:10.1289/ehp.0901339)]. Exposure to OH-PBDEs resulted in a dose-dependent increase in average and amplitude of [Ca^2+^]*_i_* with varying LOECs. LOECs are determined by the amplitude of the increase in [Ca^2+^]*_i_* (Figure 2) and the percentage of cells showing initial and late increases in [Ca^2+^]*_i_* (Figure 3). No effects were detected on the average and amplitude of [Ca^2+^]*_i_* during exposure to 20 μM 6-MeO-BDE-47, and we observed no effects of 6-MeO-BDE-47 on cell viability, the percentage of cells with increases in [Ca^2+^]*_i_* to > 175% of baseline, or the average frequency, duration, or amplitude of fluctuations.

**Figure 4:**
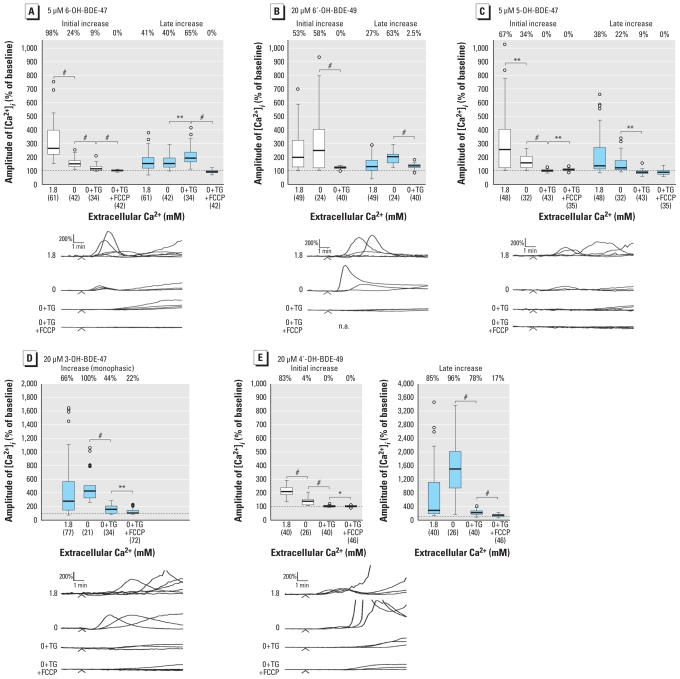
Increase in [Ca^2+^]*_i_* by OH-PBDEs results from the release of Ca^2+^ from intracellular stores. Values shown are amplitudes of [Ca^2+^]*_i_* in PC12 cells during exposure to 5 μM 6-OH-BDE-47 (*A*), 20 μM 6′-OH-BDE-49 (*B*), 5 μM 5-OH-BDE-47 (*C*), 20 μM 3-OH-BDE-47 (*D*), and 20 μM 4′-OH-BDE-49 (*E*) measured in external saline (1.8 mM Ca^2+^), Ca^2+^-free saline (0 mM Ca^2+^), Ca^2+^-free saline after pretreatment with TG (0 + TG), and Ca^2+^-free saline after pretreatment with both TG and FCCP (0 + TG + FCCP). Cells were pretreated with TG and FCCP to deplete Ca^2+^ stores in ER and mitochondria, respectively. Tops and bottoms of boxes represent upper and lower quartiles, the line within the box is the median, whiskers represent lowest and highest values, and circles represent outliers (for clarity, outliers that are more than three interquartile ranges from the boxes are not shown). Data are from three to seven experiments per treatment. Numbers shown in parentheses indicate the number of cells used for data analysis; the percentages of responding cells (with increase in [Ca^2+^]*_i_* to > 175% of baseline) are denoted above each box. Representative traces of [Ca^2+^]*_i_* measurements of individual PC12 cells exposed to OH-PBDE for 10 min (applied as indicated by arrowheads) in external saline (containing 1.8 mM Ca^2+^) and in Ca^2+^-free conditions are shown below. *p < 0.05, **p < 0.01, and #*p* < 0.001.

For 6′-OH-BDE-49, 3-OH-BDE-47, and 4′-OH-BDE-49, the LOEC for increased [Ca^2+^]*_i_* is 20 μM. Exposure to 20 μM 6′-OH-BDE-49 results in an initial increase in 50% of the cells. We observed late increases less frequently and with lower amplitude. The shapes of the increases in [Ca^2+^]*_i_* during exposure to 20 μM 6′-OH-BDE-49 vary widely (Figure 4B). We observed initial increases during exposure to 20 μM 3-OH-BDE-47 in 48% of the cells. Exposure to 20 μM 4′-OH-BDE-49 resulted in a modest initial increase compared with baseline in 83% of the cells, after which we observed a larger late increase.

For the OH-PBDEs with an effect at 2 μM (6-OH-BDE-47 and 5-OH-BDE-47), we also tested lower concentrations (Figures 2B,C, and 3B,C), showing that the LOECs for these OH-PBDEs are 1 μM. We observed late increases only at concentrations ≥ 2 μM. At concentrations ≤ 5 μM, this late increase had an amplitude comparable to that of the initial increase (~ 200–400% of baseline). At 20 μM, the late increase induced by 6-OH-BDE-47 or 5-OH-BDE-47 was much larger [see Supplemental Material, Table 2 (doi:10.1289/ehp.0901339)], which we also observed for 20 μM 4′-OH-BDE-49. The amplitudes of the initial and late increases that we observed during exposure to 6-OH-BDE-47 or 5-OH-BDE-47 were dose dependent (ANOVAs: 6-OH-BDE-47, initial *p* < 0.0001, late *p* < 0.0001; 5-OH-BDE-47, initial *p* < 0.01, late *p* < 0.0001).

The mean amplitude of [Ca^2+^]*_i_* during exposure to 20 μM of the hydroxylated metabolites was independent of the position (*ortho*, *meta*, or *para*) of the OH group on the PBDE molecule (ANOVA, not significant). However, OH-PBDEs in which the OH group was shielded on only one side, with either the other phenyl ring or a Br atom, induce significantly higher increases in [Ca^2+^]*_i_* compared with OH-PBDEs in which the OH group was shielded on both sides (ANOVA, *p* < 0.01). However, this influence of the shielding of the OH group (on one compared with two sides) was independent of its position (*ortho*, *meta*, or *para*) on the PBDE molecule (ANOVA, not significant).

### Hydroxylated BDE-47 metabolites increase [Ca^2+^]_i_ by release of Ca^2+^ from intracellular stores

Although initial increases in [Ca^2+^]*_i_* induced by 6-OH-BDE-47, 5-OH-BDE-47, and, to a lesser extent, 4′-OH-BDE-49 were reduced in Ca^2+^-free conditions, increases were still observed for all of the OH-PBDEs (Figure 4). To identify the responsible Ca^2+^ stores, endoplasmic reticulum (ER) or both ER and mitochondrial Ca^2+^ stores were depleted by either 1 μM thapsigargin (TG) or 1 μM TG plus 1 μM carbonyl cyanide 4-(trifluoromethoxy)phenylhydrazone (FCCP). TG and TG/FCCP pretreatment, respectively, caused a transient increase in [Ca^2+^]*_i_* of 321 ± 11% (*n* = 191) and 393 ± 11% of baseline (*n* = 195), after which [Ca^2+^]*_i_* stabilized and the OH-PBDE was applied.

When we depleted ER Ca^2+^ stores in Ca^2+^-free conditions, the initial increase induced by 5 μM 6-OH-BDE-47 was largely diminished (Figure 4A). Although the amplitude of the late increase was not different in Ca^2+^-free conditions, it increased after depletion of the ER Ca^2+^ stores, but greatly diminished after depletion of both ER and mitochondrial Ca^2+^ stores.

We also observed the variation in [Ca^2+^]*_i_* responses during exposure to 20 μM 6′-OH-BDE-49 in Ca^2+^-free conditions (Figure 4B). After depletion of ER Ca^2+^ stores, we no longer observed either initial or late transient increases in [Ca^2+^]*_i_*.

When the ER Ca^2+^ stores were depleted, the amplitudes of both initial and late increases induced by 5 μM 5-OH-BDE-47 were largely diminished (Figure 4C). After depletion of both ER and mitochondrial Ca^2+^ stores, the late increase was further reduced.

We observed monophasic increases during exposure to 20 μM 3-OH-BDE-47 in Ca^2+^-free conditions, with similar amplitude (Figure 4D). After depletion of the ER Ca^2+^ stores, the amplitude was significantly decreased, and even further after depletion of ER and mitochondrial Ca^2+^ stores.

During exposure to 20 μM 4′-OH- BDE-49 after depletion of the ER Ca^2+^ stores, the amplitude of the initial increase largely diminished (Figure 4E). The amplitude of the late increase was not significantly changed in Ca^2+^-free conditions. After depletion of the ER Ca^2+^ stores, the amplitude significantly decreased, and even further after depletion of ER and mitochondrial Ca^2+^ stores.

### Lower LOECs were identified for hydroxylated metabolites when investigating fluctuations

At LOECs and NOECs based on the amplitude of [Ca^2+^]*_i_*, we investigated fluctuations in [Ca^2+^]*_i_* [Table 1; also see Supplemental Material, Table 4 (doi:10.1289/ehp.0901339)]. We detected no effects on the percentage of cells showing fluctuations or the frequency, duration, and amplitude of these fluctuations during exposure to 2 μM 6′-OH-BDE-49 or 3-OH-BDE-47. During exposure to 0.2 μM 6-OH-BDE-47, we observed an increase in the percentage of cells showing fluctuations. The average duration of fluctuations increased, without effects on the frequency or amplitude (NOEC, 0.1 μM 6-OH-BDE-47). At 1 μM 5-OH-BDE-47, the number of cells showing fluctuations increased. Although the duration increased, the amplitude and frequency were not affected (NOEC, 0.2 μM 5-OH-BDE-47). At 2 μM 4′-OH-BDE-49, the number of cells showing fluctuations increased, as well as the average fluctuation frequency, duration, and amplitude (NOEC, 1 μM 4′-OH-BDE-49).

## Discussion

The OH-PBDE metabolite 6-OH-BDE-47 has previously been shown to disrupt [Ca^2+^]*_i_* in PC12 cells by releasing Ca^2+^ from intracellular stores at lower concentrations than its parent compound BDE-47 (Dingemans et al. 2008). The results presented here demonstrate that other hydroxylated metabolites of BDE-47 also induce Ca^2+^ release from intracellular stores, whereas the methoxylated analog (6-MeO-BDE-47) and the parent compounds lack this effect (Figure 2). Experiments in Ca^2+^-free conditions (Figure 4) indicate that the initial increases induced by 6-OH-BDE-47, 5-OH-BDE-47, and 4′-OH-BDE-49 are partly caused by influx of extracellular Ca^2+^. The initial and late increases induced by 6-OH-BDE-47 are caused by release of Ca^2+^ from ER and mitochondria, respectively. The widely varying increase induced by 6′-OH-BDE-49 was caused primarily by release from ER Ca^2+^ stores. Both initial and late increases induced by 5-OH-BDE-47 and the increase induced by 3-OH-BDE-47 are caused by release of Ca^2+^ mainly from ER, but also from mitochondria. Both initial and late increases induced by 4′-OH-BDE-49 are caused by release of Ca^2+^ from ER, but the late increases are also from mitochondria. When investigating fluctuations in [Ca^2+^]*_i_*, we detected subtle effects of BDE-47 on the Ca^2+^ homeostasis and observed lower LOECs for several OH-PBDEs (Table 1).

We detected no or mild effects on cell viability for the investigated PBDEs and MeO/OH-PBDEs, indicating that the observed effects on [Ca^2+^]*_i_* were not confounded by cytotoxicity. The AB assay, which is based on mitochondrial activity, appeared to be less useful in determining cell viability because for 6-OH-BDE-47, 6′-OH-BDE-49, and 3-OH-BDE-47 the relative fluorescence intensity increased dose dependently above the control level, suggesting induction of mitochondrial activity. This may be related to mitochondrial uncoupling, which was previously demonstrated for 6-OH-BDE-47 in isolated zebrafish mitochondria (van Boxtel et al. 2008).

The low basal [Ca^2+^]*_i_* of PC12 cells is maintained by the removal of Ca^2+^ ions by the plasma membrane Ca^2+^ ATPases and Na^2+^–Ca^2+^ exchanger (Duman et al. 2008; for review, see Westerink 2006). Additionally, Ca^2+^ can be sequestered into organelles, mainly ER and mitochondria. Both 3-OH-BDE-47 and 4′-OH-BDE-49 induce increases in [Ca^2+^]*_i_* even after depletion of both ER and mitochondria by TG and FCCP. Ca^2+^ has also been shown to accumulate in endosomes, lysosomes, secretory granules, the Golgi apparatus, and nucleus (for review, see Laude and Simpson 2009). The Golgi apparatus stores Ca^2+^ via sarcoplasmic/ER Ca^2+^ ATPase pumps (Missiaen et al. 2007), which are inhibited by TG. Therefore, it is unlikely that release of Ca^2+^ from the Golgi apparatus caused the additional increase, whereas this remains unclear for the other mentioned organelles.

At concentrations not affecting the average and amplitude of increases in [Ca^2+^]*_i_*, BDE-47, 6-OH-BDE-47, and 4′-OH-BDE-49 caused an increase in the frequency, amplitude, and/or duration of fluctuations in [Ca^2+^]*_i_*. These subtle effects on Ca^2+^ homeostasis resulted in lower NOECs for most of these brominated flame retardants, particularly BDE-47 (Table 1). Ca^2+^ signals vary from microdomains to globally across the cell and from milliseconds to many hours (Laude and Simpson 2009). Because the measured [Ca^2+^]*_i_* is an average value for the entire cytosol, underestimation of membrane- or store-associated high [Ca^2+^]*_i_* or high [Ca^2+^]*_i_* microdomains (for review, see Cheng and Lederer 2008) can be expected. The timing of Ca^2+^ signals, including frequency and duration, affects how external stimuli cause (patho)physiologic results (Boulware and Marchant 2008). Moreover, [Ca^2+^]*_i_* transients trigger activity-dependent developmental events in neurons, by activating gene expression, cytoskeletal elements, or neurotransmitter release, whereas the characteristics of these responses are determined by amplitude, frequency, source, and spatial location of Ca^2+^ signals (for review, see Moody and Bosma 2005). Therefore, the observed effects of OH-PBDEs at low concentrations can be of relevance for the development of the nervous system.

None of the parent PBDEs except BDE-47 showed any effects on Ca^2+^ homeostasis in PC12 cells. Nonetheless, neurotoxic effects of BDE-47, BDE-99, BDE-100, and BDE-153 have been detected at different biological levels (Costa and Giordano 2007). Because of the lack of effects by parent PBDEs in the present study, no effects of bromination pattern could be investigated. We confirmed that the activity of the OH-PBDEs depended on the presence of the OH group, because no effects were observed during exposure to the methoxylated analog of 6-OH-BDE-47. The higher activity of 6-OH-BDE-47 compared with its methoxylated analog is in line with other studies, mostly on endocrine effects (Cantón et al. 2008; Kojima et al 2009; van Boxtel et al. 2008). The mean amplitude of increases in [Ca^2+^]*_i_* could not be related to the location of the OH group on the PBDE molecule. However, it appeared that when the OH group was shielded on both sides by either the other phenyl ring and/or Br atoms (as in 6′-OH-BDE-49 and 3-OH-BDE-47), the OH-PBDE increased [Ca^2+^]*_i_* less than when the OH group was less shielded (as in 6-OH-BDE-47, 5-OH-BDE-47, and 4′-OH-BDE-49). Also, the OH-PBDEs with only one shielded side of the OH group induced release of Ca^2+^ from ER at the lowest concentrations (6-OH-BDE-47 and 5-OH-BDE-47) or with the highest amplitude in Ca^2+^-free conditions (4′-OH-BDE-49). Thus, the toxicity of OH-PBDEs appears attenuated by shielding of the OH group on both sides by either the other phenyl ring and/or Br atoms.

Several animal studies confirmed the generation of hydroxylated metabolites of PBDEs *in vivo* (Hakk et al. 2009; Malmberg et al. 2005; Marsh et al. 2006), and that OH-PBDEs were also formed in human liver cells exposed to BDE-99 (Stapleton et al. 2009). Interestingly, marine organisms have also been shown to produce hydroxylated and methoxylated metabolites (Hakk and Letcher 2003).

Only very recently, the occurrence and accumulation of hydroxylated metabolites were confirmed in humans (Athanasiadou et al. 2008), with total OH-PBDE serum concentrations up to 120 pmol/g lipids. Another study also detected OH-PBDEs in U.S. fetal serum samples and confirmed the bioaccumulation of these metabolites (Qiu et al. 2009). Moreover, they demonstrated that concentrations of OH-PBDEs were similar or sometimes even higher than the concentration of PBDEs. Fetal total OH-PBDE serum concentrations ranged from 2.01 to 899.1 ng/g lipids (median, 21.96 ng/g lipids). The most abundant BDE-47 metabolites found in fetal blood were 5-OH-BDE-47 and 6-OH-BDE-47 (Qiu et al. 2009). It is concerning that these metabolites caused an increase of [Ca^2+^]*_i_* at much lower concentrations than the other metabolites of BDE-47 investigated.

All five OH-PBDEs investigated in the present study caused Ca^2+^ release from intracellular Ca^2+^ stores, although with different LOECs. Likely, depending on the position, the OH group and adjacent phenyl ether and/or Br atoms, other hydroxylated metabolites of tetra and pentaPBDEs have a similar effect on cellular calcium homeostasis. The median (21.96 ng/g lipids) and highest (899.1 ng/g lipids) concentrations of total OH-PBDEs observed in fetal plasma correspond to approximately 0.4 and 17.4 nM, respectively, in blood (calculated with average physiologic parameters). Thus, the highest concentration observed in human blood is only two orders of magnitude lower than the LOEC for Ca^2+^ release from intracellular stores by OH-PBDEs (1 μM). Moreover, the LOEC for increased Ca^2+^ fluctuations is even lower (0.2 μM), meaning that the margin of exposure is insufficient in some individual exposure situations. Also, because OH-PBDEs are not associated with lipids—as are the parent PBDEs—but have a high affinity for plasma proteins (Verreault et al. 2005), the estimated blood concentration calculated from exposure values at a lipid-weight–adjusted basis (nanograms per gram lipids) may be underestimated. However, the LOEC (1 μM) used to calculate the margin of exposure is higher for other metabolites, and it remains to be determined whether the observed effects on fluctuations in [Ca^2+^]*_i_* could result in functional or even adverse effects *in vivo*.

Because exposure to organohalogen compounds within the time frame of rapid brain development can result in behavioral defects in mice (for review, see Costa and Giordano 2007), it is concerning that children are exposed to these environmental pollutants prenatally and postnatally. Moreover, several studies have observed interactions between environmental pollutants to enhance (neuro)toxicity. Additive and synergistic neurotoxic effects of polychlorinated biphenyls (PCBs) and PBDEs have been detected *in vivo* (Eriksson et al. 2006) and *in vitro* (Gao et al. 2009). Concern about possible effects on the developing brain arises from the fact that an increase in [Ca^2+^]*_i_* by release from intracellular stores appears to be a common mechanism for OH-PBDEs and *ortho*-PCBs (for review, see Mariussen and Fonnum 2006). Therefore, a possible additive effect of these environmental pollutants with respect to increases in cytosolic [Ca^2+^]*_i_*, which not only is a trigger for neurotransmitter release but affects many cellular processes (Clapham 2007), is not unlikely.

Because very high concentrations of PBDEs are occasionally measured in humans, the voluntary and legislative measures to reduce the release of PBDEs into the environment appear justified. Also, hydroxylated metabolites of PBDEs, which were recently found to bioaccumulate in humans (Athanasiadou et al. 2008; Qiu et al. 2009), either from man-made PBDEs or of natural origin, are currently not taken into account in regulatory human risk assessment. The results presented here reveal a structure–activity relationship for metabolites of PBDEs (more shielding of the OH group reduces the potency of OH-PBDEs) and reinforce that oxidative metabolism should be included in human risk assessment of persistent organic pollutants.

## Figures and Tables

**Table 1:** LOECs (μM) of BDE-47 and hydroxylated metabolites for cell viability and different parameters of [Ca^2+^]*_i_* in PC12 cells.

		Ca^2+^homeostasis				Fluctuations in [Ca^2+^]*_i_*				
	Decreased cell viability	Ca^2+^ influx (extracellular)	Ca^2+^ release		NOEC	Percent cells showing fluctuations	Fluctuation			NOEC
			ER	Mitochondria			Frequency	Duration	Amplitude	
BDE-47	—	—	—	—	20	—	2	2	—	1 (<)
6-OH-BDE-47	20	1	1	2	0.2	0.2	—	0.2	—	0.1 (<)
6′-OH-BDE-49	—	—	20	—	2	20^a^	20^a^	20^a^	20^a^	2^b^
5-OH-BDE-47	20	1	1	—	0.2	1	—	1	—	0.2^b^
3-OH-BDE-47	—	—	20	20	2	20^a^	20^a^	20^a^	20^a^	2
4′-OH-BDE-49	20	20	20	20	2	2	2	2	2	1 (<)

—, not detected. For the mechanisms responsible for effects on Ca^2+^ homeostasis, under “Ca^2+^ homeostasis,” values shown are LOEC for effects causing a decrease of > 25% in the specific Ca^2+^-free experiments (Figure 4), and resulting NOEC levels. For the investigated parameters of fluctuations in [Ca^2+^]*_i_*, under “fluctuations in [Ca^2+^]*_i_*,” values shown are LOEC and resulting NOEC levels; NOEC levels for effects on fluctuations in [Ca^2+^]*_i_* that are lower than NOEC values for effects on Ca^2+^ homeostasis-related processes are indicated by (<). For values of [Ca^2+^]*_i_* and fluctuation parameters, see Supplemental Material, Tables 2 and 3 (doi:10.1289/ehp.0901339). For all PBDEs and MeO/OH-PBDEs investigated in this study, see Supplemental Material, Table 4.

a Possible effects on parameters of fluctuations are obscured by release of Ca^2+^ from intracellular stores.

b The number of data points available to investigate duration and amplitude of the fluctuations in [Ca^2+^]*_i_* is insufficient to ensure the NOEC.
